# Comparative Morphology of the *Papillae Linguales* and their Connective Tissue Cores in the Tongue of the Greater Japanese Shrew-mole, *Urotrichus talpoides*

**DOI:** 10.1111/j.1439-0264.2012.01159.x

**Published:** 2012-05-10

**Authors:** K Yoshimura, J Shindo, I Kageyama

**Affiliations:** 1 Department of Anatomy, Faculty of Life Dentistry, The Nippon Dental University at Niigata1–8 Hamaura-cho, Chuo-ku, Niigata City, Niigata, 951-8580, Japan; 2 Laboratory of Wildlife Science, Department of Environmental Bioscience, School of Veterinary Medicine, Kitasato UniversityHigashi 23-35-1, Towada, Aomori, 034-8628, Japan

## Abstract

The external morphology of the *papillae linguales* (*papillae filiformes*, *papillae fungiformes* and *papillae vallatae*) and their connective tissue cores (CTCs) of the greater Japanese shrew-mole (*Urotrichus talpoides*) were analysed by optical and scanning electron microscopy. *Papillae filiformes* were distributed over the dorsal surface of the apex linguae, and on the rostral and caudal regions of the corpus linguae but were less numerous in the mid-region. They were absent from the radix linguae. A pair of oval *papillae vallatae* was situated at the border between the corpus linguae and the radix linguae. *Papillae foliatae* were absent. The epithelial surface of each *papilla filiformis* consisted of a circular concavity, a ring-like wall and either a single thumb-like process or 2–3 slender pointed processes, depending on their location. The morphology of the CTCs of the *papillae filiformes* also varied regionally. The *papillae linguales* of the Japanese shrew-mole were morphologically similar to those of other Talpidae and Soricidae*,* including the common shrew, particularly with respect to the *papillae filiformes* in the mid- and caudal regions of the corpus linguae.

## Introduction

The mammalian family Talpidae (moles and shrew-moles), within the order Soricomorpha (shrews), contains well-known living representatives. They were formerly placed within the ‘Insectivora’ but the systematics of this grouping is problematic, particularly with respect to the position of the poorly known Asiatic (Japanese) shrew-moles (Symonds, 2005). Molecular phylogenetic studies (Tsuchiya et al., 2000; Nikaido et al., 2003; Shinohara et al., 2003) have shown that the Japanese shrew-mole (*Urotrichus*) was phylogenetically distant from European and Japanese moles (*Talpa* and *Mogera*, respectively). Consequently, the systematics of the subfamilies of the Talpidae remains controversial (Wilson and Reeder, 2005).

Many studies have demonstrated morphological diversity among the oral tissues of mammals (Nickel et al., 1979), particularly with respect to the three-dimensional structure of the *papillae linguales* and their connective tissue cores (CTCs) in terrestrial species (Kobayashi et al., 1989, 2005; Kobayashi, 1992; Adnyane et al., 2011; Watanabe et al., 2011). Following the early macroscopic studies of insectivores by Sonntag (1923), there have been several detailed morphological investigations into the *papillae linguales* of the Soricomorpha, for example, Talpidae such as *Mogera* (Kobayashi et al., 1983; Miyata et al., 1990), *Talpa* (Jackowiak, 2006) and *Dymecodon* (Kobayashi et al., 1983); and Soricidae including *Solex*, (Kobayashi and Iwasaki, 1989; Jackowiak et al., 2004), *Suncus* (Kobayashi et al., 1983; Kobayashi and Iwasaki, 1989), *Chimarrogale* (Kobayashi and Iwasaki, 1989) and *Crocidura* (Kobayashi (1992), Kobayashi et al. (1989, 2005). However, information is lacking for the *papillae linguales* and for their underlining CTCs of the Japanese shrew-moles (*Urotrichus)*.

The aim of this study was to analyse in detail the surface morphology of the *papillae linguales* on the dorsal surface of the tongue of the Japanese shrew-mole and, after exfoliation of the epithelium of their CTCs, to compare them with earlier descriptions of Talpidae and other Soricomorpha.

## Materials and Methods

### Animals and tissue preparation

Eight Japanese shrew-moles *Urotrichus talpoides* (three males, body length 10.3–10.8 cm, 12.5–15.0 g; five females, 10.2–10.8 cm, 12.3–15.5 g) that had died in road accidents were used for this study. They were fixed with 10% formalin for post-mortem autopsy and, shortly afterwards, tissue blocks were excised from various regions of the tongue.

### Light microscopy

Tissue samples were dehydrated with a graded ethanol series, embedded in paraffin wax and sectioned at 4 μm. Sections were stained with haematoxylin–eosin and observed by bright-field microscopy (BH-2; Olympus, Tokyo, Japan).

### Scanning electron microscopy

Tissue samples were immersed in 3.5 N HCl for 5 days at room temperature (25–28°C). The epithelium was then exfoliated from the underlying CTCs. Specimens were washed with tap water and treated with a 0.5% tannic acid solution. Post-fixation was accomplished by immersion for 10 min in 1% OsO_4_. The tissue was then washed and dehydrated with a graded ethanol series.

After dehydration, specimens were freeze-dried using t-butyl alcohol (Inoue and Osatake, 1988), coated with Pt-Pd and observed with a scanning electron microscope (SEM) (S-800; Hitachi-Hi-Technologies, Tokyo, Japan).

## Results

### Macroscopic overview

**Fig. 1:**
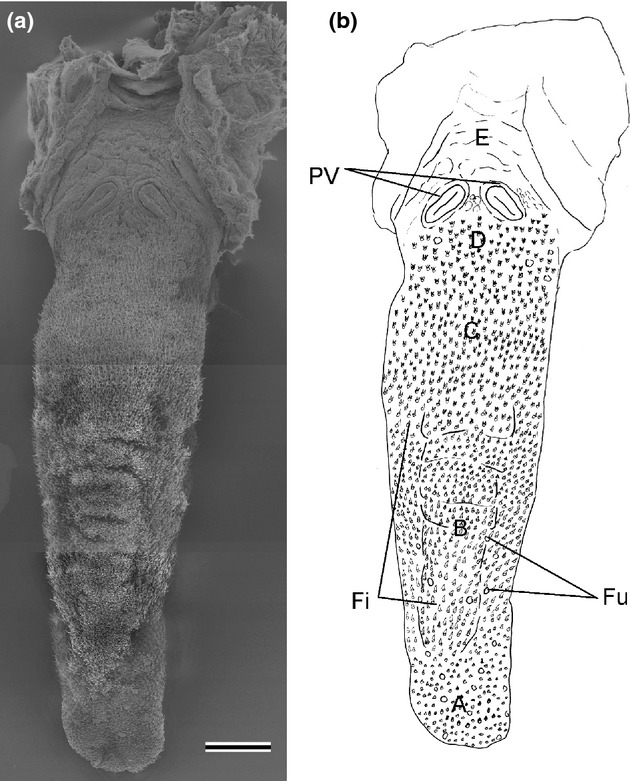
(a) Overview of the dorsal surface of the tongue of the greater Japanese shrew-mole *Urotrichus talpoides*. Scale bar = 1000 μm, (b) Diagram of the tongue. A, apex linguae; B, rostral; C, middle; and D, caudal regions of the corpus linguae; E, radix linguae; Fu, *papillae fungiformes*; Fi, *papillae filiformes*; PV, *papillae vallatae*.

Macroscopically, the tongue of the greater Japanese shrew-mole (Fig. 1) was elongated in rostro–caudal direction, with a rounded apex linguae. No torus linguae were present. Numerous *papillae filiformes* (Fig. 1; Fi) were distributed over the entire dorsal surface of the tongue except on the radix linguae. *Papillae fungiformes* (Fig. 1; Fu) were scattered over the apex linguae (Fig.1b; A) and on the rostral (Fig.1b; B) and caudal regions (Fig.1b; D) of the corpus linguae but they were fewer in the mid-region (Fig.1b; C). Neither a sulcus medianus linguae nor a sulcus terminalis linguae were present. A pair of oval or rostrolaterally obliquely elongate *papillae vallatae* (Fig. 1b; PV) was situated at the boundary between the caudal section (Fig.1b; D) of the corpus linguae and the radix linguae (Fig.1b; E). *Papillae foliatae* and lateral organ-like structures (Sonntag, 1925) were absent. At the caudal end of the *radix linguae* (Fig.1b; E), the *papillae linguales* were attenuated, forming only weak folds.

### Microscopic observation

#### Papillae filiformes

**Fig. 2:**
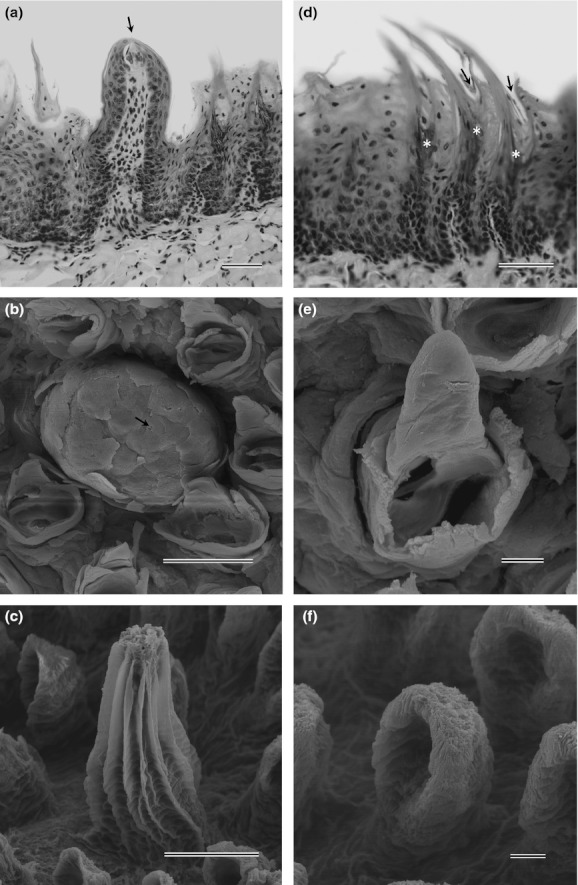
A set of *papillae linguales* distributed on the apex and/or rostral part of the corpus. (a) Sagittal histological section of *papillae fungiformes* situated on the apex linguae (sagittal section). Taste buds (indicated with arrows) are present on the top of the papilla. Scale bar: 50 μm. (b) SEM micrograph of the external surface of *papillae fungiformes* distributed on the apex linguae. Taste pore (arrow) is present on the dome-like *papilla fungiformis*. Scale bar: 50 μm. (c) SEM micrograph of the connective tissue core (CTC) of a *papilla fungiformis* situated on the apex linguae, after epithelial exfoliation. The conical CTC has numerous frill-like processes. Scale bar: 50 μm. (d) Sagittal histological section of *papillae filiformes* distributed on the apex linguae. The stratum corneum of the *papillae filiformes* is thick and sharply pointed. Concavities (indicated as arrows) are situated in front of each papilla. Scale bar: 50 μm. (e) SEM micrograph of the apex linguae representing the epithelial surface of *papilla filiformis*. The thick conical processes of *papillae filiformes* are inclined caudally. A concavity surrounded by a ring-like process is present in front of each papilla. Scale bar: 10 μm. (f) CTCs of the *papillae filiformes* distributed on the *apex linguae* after the removal of the epithelium. A rounded core with a hemispherical concavity is observed in front of the papilla. Scale bar: 10 μm.

Light microscopy revealed that the epithelium of *papillae filiformes* (Fig. 2d) was keratinized, especially on the caudal aspect. A concavity was present rostral to each papilla (Fig. 2d, 4a; arrows). Keratohyalin granules were observed in the apex linguae and in the caudal parts of the papillae (Fig. 2d, 4a). The *papillae filiformes* on the apex linguae were more erect than those in the caudal regions of the corpus linguae, where the *stratum granulosum* containing keratohyalin granules exhibited a compressed appearance. A number of nuclei were present in superficial cells of the interpapillary epithelium throughout the dorsal surface of the tongue. In sagittal sections, the lamina propria of the CTCs of the *papillae filiformes* had a different appearance in different regions of the tongue. On the apex linguae, the CTCs were rounded and extended only into the lower third of the epithelium. In contrast, those in the caudal section of the corpus linguae had pointed tips and extended almost into the superficial epithelial layer.

**Fig. 3:**
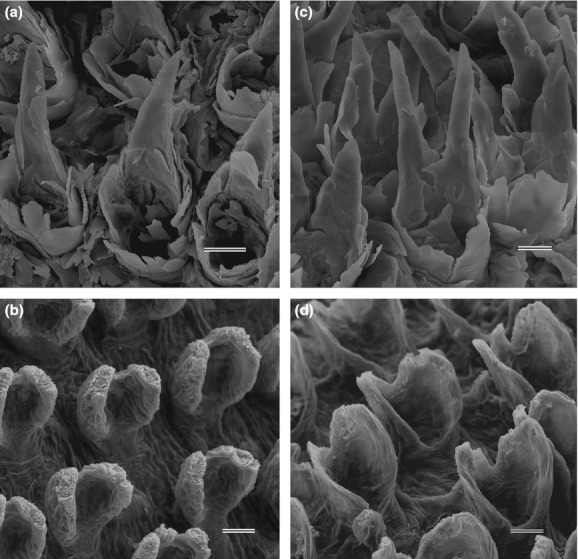
A set of *papillae filiformes* distributed on the rostral (see Fig. 1b, B) or middle (see Fig.1b, C) part of the corpus linguae. (a) SEM micrograph of the epithelial surface of *papillae filiformes* in the rostral region of the corpus. The *papillae filiformes* in this area exhibit sharp main processes. Scale bar: 20 μm. (b) SEM micrograph of the CTCs of the *papillae filiformes* of the rostral part of the corpus after the removal of the epithelium. The CTCs of *papillae filiformes* in this area possess a notch at the centre of the CTC. Scale bar: 20 μm. (c) SEM micrograph of the epithelial surface of *papillae filiformes* situated in the middle region of the corpus. The *papillae filiformes* in this area have two sharp main processes. Scale bar: 20 μm. (d) SEM micrograph of the CTCs of the *papillae filiformes* in the middle region observed after epithelial exfoliation. The edges of the CTCs of *papillae filiformes* are thin and possess a deep cleft in the midline. Scale bar: 20 μm.

**Fig. 4:**
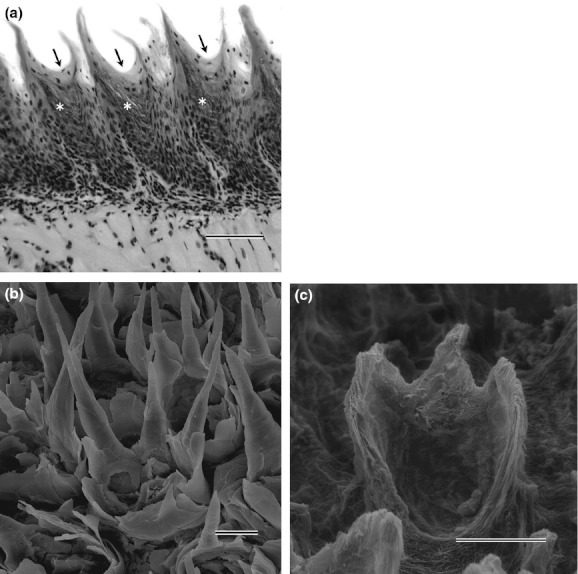
A set of *papillae filiformes* distributed on the caudal section of the corpus linguae (see Fig. 1b, D). (a) Sagittal histological section of *papillae filiformes* on the caudal area of the corpus. Goblet-like concavities (arrows) are situated in front of each papilla. Numerous keratohyalin granules (asterisk) are present in the stratum corneum and appear to be located beneath the concavities. Scale bar: 60 μm. (b) SEM micrograph of the epithelial surface of *papillae filiformes* situated on the caudal area of the corpus. *Papillae filiformes* distributed in this area have three sharp main processes. Scale bar: 30 μm. (c) SEM micrograph of the CTC of the *papillae filiformes* at the caudal area of the corpus linguae after the removal of the epithelium. CTCs of *papillae filiformes* in this area possess two notches. A cradle-like concavity is situated in front of the CTC of *papillae filiformes*. Scale bar: 30 μm.

Visualization of the external surface by scanning electron microscopy revealed that each *papilla filiformis* was associated with a round concavity (Fig. 2d and e, Fig. 3a and c, Fig. 4b) and was surrounded by a ring-like wall. On the apex linguae, a thumb-like conical process (Fig. 2e) was situated on the caudal part of the rim of each *papilla filiformis* and was inclined caudally. The appearance of the *papillae filiformes* varied depending on the area. In the rostral section of the corpus linguae (Fig. 1b), the processes of the *papillae filiformes* were somewhat elongated vertically, with a sharp tip (Fig. 3a). *Papillae filiformes* distributed over the middle of the corpus (Fig. 1b) possessed two sharp processes (Fig. 3c), whereas in the caudal region (Fig. 1b), there were three slender sharp conical processes (Fig. 4b). After exfoliation of the epithelium, the CTCs of the *papillae filiformes* reflected the morphological differences of the epithelial surface. Each CTC of the *papillae filiformes* on the apex linguae (Fig. 1b) consisted of a single, smooth, dome-like protuberance with a hemispherical depression on the rostral surface. CTCs that were distributed on the rostral section of the corpus (Fig. 1b) had a cleft at the top (Fig. 3b). In the middle section (Fig. 1b), the CTCs were sharp-edged with a V-shaped cleft (Fig. 3d). Furthermore, CTCs in the caudal region of the corpus (Fig. 1b) possessed two clefts (Fig. 4c), presumably reflecting their overlying epithelial processes. The *papillae filiformes* were approximately 50–170 μm long and 35–86 μm wide.

#### Papillae fungiformes

Under the light microscope, the apices of the *papillae fungiformes* (Fig. 2a) were dome-like. Keratinization of these papillae was weak and some of the more superficial epithelial cells retained nuclei. A few taste buds were observed in the epithelium at the tips of the *papillae fungiformes*.

In SEM observations, the external surface of *papillae fungiformes* distributed on the rostral part of the corpus linguae (Fig. 2b) was smooth and dome-like, although their size varied. After epithelial exfoliation, the CTCs of *papillae fungiformes* on the apex linguae (Fig. 2c) were columnar with several vertically aligned, narrow ridges. *Papillae fungiformes* were approximately 78–148 μm in diameter.

#### Papillae vallatae

**Fig. 5:**
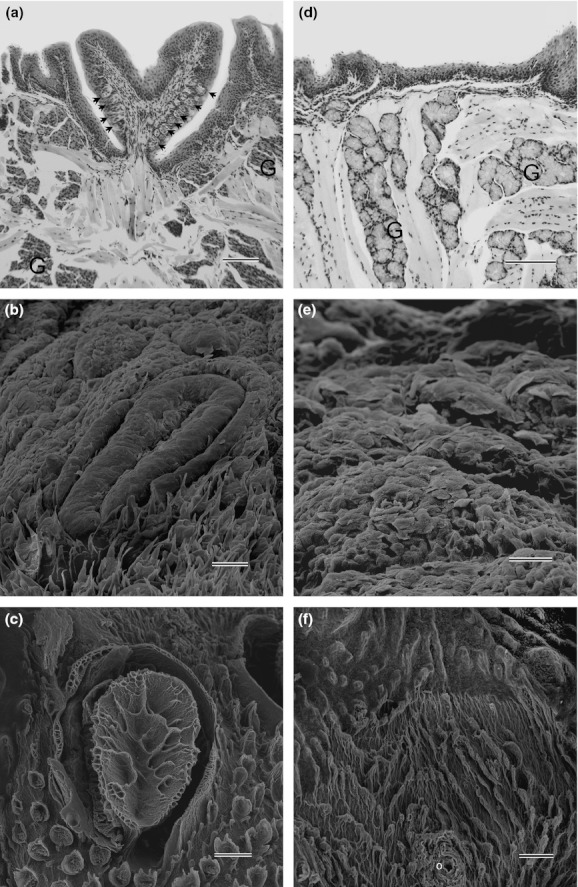
A set of *papillae linguales* distributed on the radix linguae (see Fig. 1b, E). (a) Transverse histological section of a *papilla vallata*. Numerous taste buds (arrows) are present in the inner wall of the epithelium of the peripapillary furrow. Glandulae linguales (G) are located in the lamina propria. A concavity is present in the centre of the papilla. Scale bar: 100 μm. (b) SEM micrograph of the epithelial surface of a *papilla vallata*. *Papillae vallatae* are obliquely elongated, and a cleft-like concavity is present at the tip of the papilla. Scale bar: 100 μm. (c) SEM micrograph of the *papillae vallatae* after exfoliation of their epithelium. A wall-like CTC of the furrow of a *papilla vallata* is recognizable. Numerous CTCs of *papillae filiformes* are also distributed in this area. However, each CTC of the *papillae filiformes* has many notches with a flame-like appearance. Scale bar: 100 μm. (d) Sagittal histological section of the radix linguae. Mucus-rich mixed glands of glandulae linguales (G) are situated deep in the lamina propria. Scale bar: 100 μm. (e) SEM micrograph of the epithelial surface of the radix linguae. The surface is smooth and hilly. Scale bar: 50 μm. (f) SEM micrograph of the caudal section of the radix linguae after the removal of the epithelium. Numerous thumb-like or ridge-like CTCs are distributed on the surface. Some appear to be arranged in rows. An orifice of the glandulae linguales (o) can be seen. Scale bar: 50 μm.

A pair of *papillae vallatae* (Fig. 1b) was situated at the boundary between the caudal section of the corpus (Fig.1b) and the radix linguae (Fig.1b). In the light microscope, numerous taste buds were observed in the inner epithelial wall of the circumferential furrow. Glandulae linguales were seen in the lamina propria (Fig. 5a) with their orifices opening into the circumferential groove. A concavity was present at the top of each *papilla vallata*.

In SEM preparations, the external surfaces of the *papillae vallatae* (Fig. 5b) were oval, or obliquely elongated in a rostrolateral direction. The dome-like papilla was surrounded by a circumferential ridge and furrow. Cleft-like grooves were seen at the top of the elongated papillae. After exfoliation of the epithelium, CTCs of *papillae vallatae* were concave and surrounded by a wall-like core in the circumferential furrow (Fig. 5c). *Papillae vallatae* were approximately 337–703 μm in diameter.

#### Radix linguae

The radix linguae exhibited a weakly folded appearance (Fig. 1b). Under the light microscope, large mucus-rich mixed glands of glandulae linguales were seen in the lamina propria. After the removal of the epithelium, the surface of the CTCs of the radix linguae exhibited numerous processes. Orifices of glandulae linguales were observed among the CTC processes.

## Discussion

Our observations indicate that the tongue of the Japanese shrew-mole possesses an assortment of morphological characteristics, representing features of both the Talpidae (moles and shrew-moles) and other Soricomorpha (shrews). Table 1 compares the morphological characteristics that we observed in the Japanese shrew-mole with those reported in previous investigations on other species.

**Table 1:** Comparison of morphological features of Japanese shrew-mole and other talpid and soricomorphan tongues

			*Papillae Linguae*										
				
	Macroscopic view		*Papillae Filiiformes*				*Papillae Fungiformes*						
						
Species	Apex	Corpus	Apical	Rostral	Middle	Caudal	Apical	Rostral	Middle	Caudal	*Papillae Foliatae*	*Papillae Vallatae*	*Radix linguae*
*Talpidae*													
Japanese shrew-mole													
(Present study)	Ro	Not tapered	One thumb-like	One sharp conical	Bifid	Trifid	Sp Ma/Med	Ma/Med	Few	Ma/Med	No	2:C	Fo
Furry-snouted mole													
(Kobayashi et al., 1983)	Ro	Not tapered	One conical	One conical	Bifid	Trifid	Few	Ma/Med	Ma/Med	Ma/Med	No	2:C	–
European mole													
(Jackowiak, 2006)	Ro	Not tapered	One conical	One conical	One conical	One conical	Ma	Ma Row7–10	Ma Row7–10	Ma/Med	No	2:C	CP
Large Japanese mole													
(Kobayashi et al., 1983)	Ro	Not tapered	One conical	One conical	One conical	One conical	Sp Ma/Med	Ma	Ma	Sp Ma/Med	No	2:C	CP
Small Japanese mole^a^													
(Miyata et al., 1990)	Ro	Not tapered	One conical	One conical	One conical	One conical	Ma	Ma 10	Ma 10	Ma/Med	No	2:C	CP
*Soricidae*													
Long-clawed shrew													
(Kobayashi et al., 1989, 2005; Kobayashi, 1992)	Ro	Tapered	One conical	Bifid	Bifid	Trifid	Ma Row	Ma Row	Ma Row	Ma Row	No	2:d2	–
Shinto shrew													
(Kobayashi et al., 1989, 2005; Kobayashi, 1992)	Ro	Tapered	One conical	One conical	Bifid	Trifid	Ma Row	Ma Row	Ma Row	Ma Row	No	2:d2	–
Common shrew													
(Jackowiak et al., 2004)	Po	Tapered	One conical	One conical	Bifid	Trifid	Ma Row	Ma Row	Ma Row	Ma Row	No	2:d2	–
Japanese water shrew													
(Kobayashi et al., 1989, 2005; Kobayashi, 1992)	Po	Not tapered	One conical	One conical	Bifid	Bifid	Ma	Ma1/4	Ma1/4	Sp Ma/Med	No	2:d1	Wa
House musk shrew													
(Kobayashi et al., 1983)	Po	Not tapered	One conical	One conical	Bifid	Trifid	Ma	Ma1/4	Ma1/4	Sp Ma/Med	No	2:C	–
Dsinezumi shrew													
(Kobayashi et al., 1989, 2005; Kobayashi, 1992)	Po	Not tapered	One conical	One conical	Bifid	Trifid	Ma	Ma1/4	Ma1/4	Sp Ma/Med	No	2:C	–

a Possibly a misidentification of the small Japanese mole.

ro, rounded; po, pointed; sp, sparse; Ma, marginal; Med, medial; Ma10, ten papillae distributed marginally; row, evenly spaced on the margin; Ma1/4, scattered over a quarter of the margo linguae; No, *papillae foliatae* not observed; 2, two *papillae vallatae* present; C, circular; d1, furrow with a single discontinuity; d2, furrow with two discontinuities; CP, *papillae conicae* on the radix linguae; Fo, weak folds; -, flat; Wa, wart-like processes.

First, it may be noted that the Japanese shrew-mole and the furry-snouted mole (Kobayashi et al., 1983) possess bifid and/or trifid *papillae filiformes*, whereas other talpids, including the European mole (Jackowiak, 2006), the large Japanese mole (Kobayashi et al., 1983) and the small Japanese mole (Miyata et al., 1990), possess only simple conical *papillae filiformes*. In contrast, bifid and trifid *papillae filiformes* are widely distributed on the tongue of the shrew.

The distribution patterns of *papillae fungiformes* are variable, especially among the Talpidae. In the Japanese shrew-mole, *papillae fungiformes* were sparsely distributed marginally and/or medially in the apex, which is similar to the pattern in the large Japanese mole (Kobayashi et al., 1983). In the rostral section of the tongue of the Japanese shrew-mole, *papillae fungiformes* were located marginally and medially, as they are in the furry-snouted mole (Kobayashi et al., 1983). Furthermore, few *papillae fungiformes* were present in the middle section of the tongue in the Japanese shrew-mole, which differs from the arrangement in other Talpidae and in Soricidae. In addition, the radix linguae of the Japanese shrew-mole exhibited weak folds, a feature not reported in other species.

Three types of *papillae vallatae* are present among the Soricomorpha (shrews): a circular type and two discontinuous types. The *papillae vallatae* of the Talpidae (mole and shrew-moles), including the present species, appeared to be only of the circular type.

Morphological information concerning the CTCs beneath the epithelium of the *papillae linguales* of Soricomorpha is limited and exists only for the Japanese mole (Miyata et al., 1990) and the house musk-shrew (Kobayashi et al., 1989, 2005; Kobayashi, 1992). However, both species exhibit different combinations of morphological traits with respect to the lingual papillae. In Japanese shrew-moles, we observed that the CTC of the *papillae filiformes* on the apex linguae consisted of a single dome, each with a hemispherical indentation on the rostral aspect. Another talpid, the Japanese mole (Miyata et al., 1990) has rather similar CTCs, described as ‘wooden spoon-like’, but the top edge of these CTC was somewhat thinner. In contrast, the CTCs of *papillae filiformes* (Kobayashi et al., 1989, 2005; Kobayashi, 1992) on the apex of the tongue of the house musk-shrew are spherical processes upon which a shallow groove runs in the rostrocaudal direction. Furthermore, the CTCs of *papillae filiformes* on the caudal region of the corpus of the Japanese shrew-moles possessed three clefts (trifids), whereas in the Japanese mole, the CTCs of the *papillae filiformes in this region* are vertically elongated and more conical (Miyata et al., 1990). This, therefore, represents a morphological trait that is rather different from that of the Japanese shrew-mole, despite the similarity of the epithelial surfaces of their *papillae filiformes*. Unfortunately, most previous reports of the morphology of the *papillae linguales* in Soricomorpha only provide information on their epithelial surface so that elucidation of their morphological traits is limited. As it is clear that CTCs also exhibit morphological differences, particularly those of the *papillae filiformes*, a more detailed analysis of the CTCs of other insectivores is greatly needed.

The above-mentioned morphological differences are presumably influenced by the animals' dietary habits. Most shrews are carnivorous but may also take carrion. They are primarily insectivorous, but some also eat seeds, nuts and other plant material. The diet of moles consists largely of earthworms, beetles and fly larvae and, when available, slugs (Rudge, 1968; Macdonald, 1984). In both groups of animals, therefore, the principal food consists of insects but shrews also ingest plant materials. The diet of the Japanese shrew-mole comprises mostly insects, spiders, centipedes and earthworms (Komiya, 2002; Abe et al., 2005). It is noteworthy, therefore, that the bifid or trifid morphology of the *papillae filiformes* in Japanese shrew-moles was similar to that of shrews, despite their diet being identical with other moles. This implies that the morphological trait exhibited by the Japanese shrew-moles may have been influenced more by their evolutionary history than by the environment. Thus, the exceptional morphological traits shown by the *papillae filiformes* of the Japanese shrew-mole reflect their transitional taxonomic status.

Further studies, including both morphological- and molecular-based investigations, are required to more fully elucidate the basis of the morphological specializations of the *papillae linguales* of the Japanese shrew-mole.
